# STEADI-RX: ADAPTING CDC’S STEADI INITIATIVE FOR USE IN COMMUNITY PHARMACIES

**DOI:** 10.1093/geroni/igz038.3154

**Published:** 2019-11-08

**Authors:** Elizabeth R Burns, Stefanie Ferreri, Jessica Robinson, Chelsea Refroe

**Affiliations:** 1 Centers for Disease Control and Prevention, National Center for Injury Prevention and Control, Division of Unintentional Injury, Atlanta, Georgia, United States; 2 University of North Carolina Eshelman School of Pharmacy, Chapel Hill, North Carolina, United States; 3 UNC Eshelman School of Pharmacy, Chapel Hill, North Carolina, United States; 4 University of Tennessee Health Science Center College of Pharmacy, Memphis, Tennessee, United States

## Abstract

Falls among older adults (age 65 and older) cause roughly 3 million emergency department visits and $50 billion in medical costs annually. The Centers for Disease Control and Prevention (CDC) developed the Stopping Elderly Accidents, Deaths, and Injuries (STEADI) initiative to increase clinical fall prevention. STEADI encourages screening for fall risk annually, assessing modifiable risk factors, and intervening to reduce risk using effective strategies like medication management. Medication management is critical given new data on the increased use of medications linked to falls. In 15 years, opioid use among older adults increased from 15% to 35% and anticonvulsant use tripled from 4% to 14%. To address the issue, CDC partnered with the University of North Carolina who 1) adapted STEADI tools for use in community pharmacies and 2) piloted and assessed their use in 31 North Carolina pharmacies. The adapted tools and processes were branded STEADI-Rx. Community pharmacists following the STEADI-Rx initiative, screen older patients for fall risk in the pharmacy, perform a medication review to identify medications that could increase fall risk, and intervene to reduce risk by sharing information with the patients’ healthcare providers. Providers can then optimize medications to reduce fall risk and improve health. STEADI-Rx tools include 1) a community pharmacy algorithm, outlining how pharmacists can conduct fall risk screening, assessment, and care coordination; 2) a community pharmacy fall risk checklist; and 3) communication materials to help pharmacists share information with patients’ providers. STEADI-Rx was launched in July 2019. More information is available at www.cdc.gov/STEADI.

